# Tubular g-C_3_N_4_/carbon framework for high-efficiency photocatalytic degradation of methylene blue†

**DOI:** 10.1039/d1ra02918e

**Published:** 2021-05-21

**Authors:** Haicheng Li, Linlin Zang, Fengtong Shen, Libin Wang, Liguo Sun, Fulong Yuan

**Affiliations:** School of Chemical Engineering and Materials, Heilongjiang University Harbin 150080 P. R. China sunliguo1975@163.com yuanfulong@hlju.edu.cn; State Key Laboratory of Urban Water Resource and Environment, School of Environment, Harbin Institute of Technology Harbin 150090 P. R. China

## Abstract

The preparation of high-efficiency, pollution-free photocatalysts for water treatment has always been one of the research hotspots. In this paper, a carbon framework formed from waste grapefruit peel is used as the carrier. A simple one-step chemical vapor deposition (CVD) method allows tubular g-C_3_N_4_ to grow on the carbon framework. Tubular g-C_3_N_4_ increases the specific surface area of bulk g-C_3_N_4_ and enhances the absorption of visible light. At the same time, the carbon framework can effectively promote the separation and transfer of charges. The dual effects of static adsorption and photodegradation enable the g-C_3_N_4_/carbon (CNC) framework to quickly remove about 98% of methylene blue within 180 min. The recyclability indicates that the tubular g-C_3_N_4_ can stably exist on the carbon framework during the photodegradation process. In the dynamic photocatalytic test driven by gravity, roughly 77.65% of the methylene blue was degraded by the CNC framework. Our work provides an attractive strategy for constructing a composite carbon framework photocatalyst based on the tubular g-C_3_N_4_ structure and improving the photocatalytic performance.

## Abstract 2

Tubular g-C_3_N_4_ grown on a carbon framework increased the surface area of bulk g-C_3_N_4_, enhanced the absorption of visible light and promoted the photocatalytic performance.

## Graphical abstract

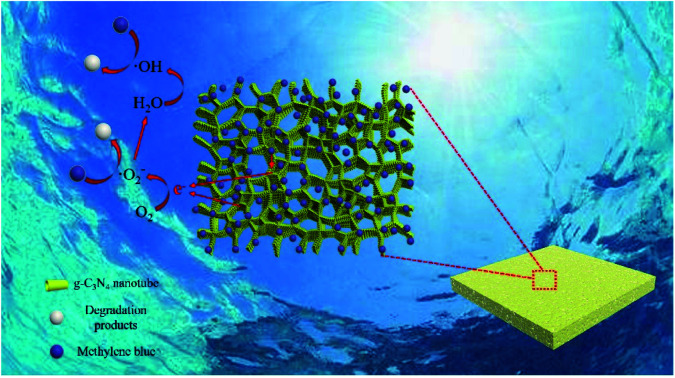

## 1 Introduction

Toxic organic dyes are difficult to degrade, so they can cause pollution to the water environment and even threaten human health. Studies have shown that photocatalytic degradation is an effective and environmentally friendly water purification method, and it has great application potential in the treatment of toxic dye wastewater.^1,2^ Considering the safety of use and possible secondary pollution, non-metallic photocatalysts have attracted more and more attention in water treatment and have potential application prospects.^3,4^

Graphitized carbon nitride (g-C_3_N_4_), as a non-metallic photocatalyst, has attracted more and more attention due to its suitable band gap (2.7 eV),^5,6^ non-toxicity, and chemical stability.^7,8^ Bulk g-C_3_N_4_ can be easily obtained by thermally induced polymerization of cyanamide,^9^ dicyandiamide,^10^ melamine,^11^*etc.* However, the aggregated bulk g-C_3_N_4_ has a small specific surface area and photo-generated carriers are easy to recombine, which to some extent hinders the charge transfer process at the g-C_3_N_4_ interface and reduces the light-collecting ability.^12,13^ In recent years, in order to improve the photocatalytic activity of g-C_3_N_4_, a variety of strategies have been reported such as construction of heterojunctions,^14^ element doping^15,16^, graphitic-carbon nitride quantum dots (g-C_3_N_4_ QDs),^17^ and g-C_3_N_4_ with unique defects.^18,19^

In order to effectively separate and transfer the photogenerated electrons of g-C_3_N_4_, combining g-C_3_N_4_ with carbon-based materials with high stability and electron transport ability has been reported to significantly increase the electron transfer of g-C_3_N_4_ during the photocatalytic process.^20,21^ For example, after being loaded on frameworks and fiber materials as the carrier,^22–24^ g-C_3_N_4_ can not only be firmly fixed on the surface of the carrier, but also can maintain its shape after photocatalysis. Such composite photocatalysts are easy to recycle and reuse, and greatly reduce operating costs. In addition, recent studies have shown that the surface morphology with a tubular structure increases the specific surface area of g-C_3_N_4_, while also exposing more active sites of the photocatalyst, thereby improving the photocatalytic performance.^25–29^

In this paper, we use waste biomass material, that is, porous carbonized grapefruit peel, as a support carrier to improve the overall conductivity of the catalyst. Through chemical vapor deposition (CVD), the tubular g-C_3_N_4_ can be *in situ* polymerized on the carbon framework. On the one hand, this method avoids the formation of bulk g-C_3_N_4_ to increase the contact area with pollutants in the water. On the other hand, it can effectively adjust the morphology of g-C_3_N_4_ by controlling the quality of the precursor to obtain ideal photocatalysts.^30–32^ Compared with bulk g-C_3_N_4_, tubular g-C_3_N_4_/carbon (CNC) framework can provide more active sites for redox reactions and inhibit the recombination of photogenerated electrons and holes, thus exhibiting strong photocatalytic activity under visible light irradiation. The result indicates that our research has great potential in the field of environmental remediation.

## 2 Experimental

### 2.1 Materials and characterization

#### 2.1.1 Chemicals and reagents

Urea and methylene blue (MB) were purchased from Sino Pharm Holding Chemical Reagent Co., Ltd. The grapefruit peel is purchased on Taobao.com. Disodium ethylenediamine tetraacetate (EDTA-2Na), isopropanol (IPA) and *p*-benzoquinone (BQ) were purchased from Sino Pharm Holding Chemical Reagent Co. All drugs are analytically pure and can be used without further purification.

#### 2.1.2 Preparation of carbon framework

**Fig. 1:**
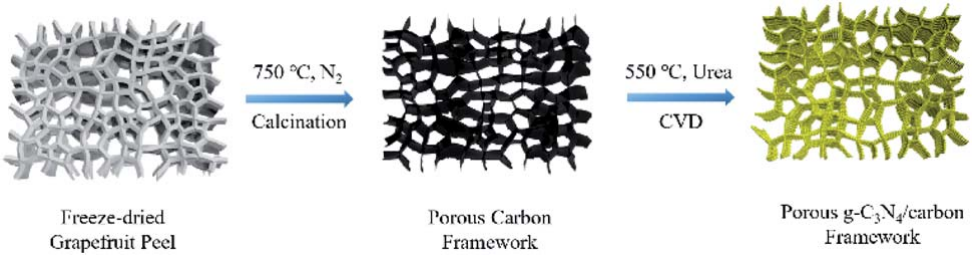
Schematic diagram of the preparation of porous g-C_3_N_4_/carbon (CNTC) framework.

The fresh grapefruit peel is freeze-dried for at least 24 h as shown in Fig. 1. Then it was put into a tubular muffle furnace and heated to 750 °C at a heating rate of 3 °C min^−1^ under nitrogen flow and kept for 2 h. Then the resulting black carbon framework was cut into 4 × 4 × 0.5 cm pieces (0.3 g).

#### 2.1.3 Preparation of g-C_3_N_4_/carbon framework

A certain amount of urea powder is poured into the bottom of the alumina crucible, and the carbon framework is placed at a height of 1 cm from the surface of the urea. Then, the crucible containing samples was heated at 550 °C for 2 h to carry out CVD process. The prepared samples are named CNC-10, CNC-12 and CNC-14, corresponding to the usage amount of urea powder of 10 g, 12 g, 14 g, respectively. For the preparation of the bulk g-C_3_N_4_, the same preparation procedure was performed without the carbonized framework.

### 2.2 Characterization

The morphology of the samples was examined through a scanning electron microscope (Hitachi, S-4800). ATR-FTIR spectra were measured using a Spectrum One instrument (Perkin Elmer, USA). An X-ray diffraction (XRD) spectroscopy analysis was performed with a Bruker D8 Advance device with a Cu Kα X-ray source. X-ray photoelectron spectroscopy was carried out using ESCALAB 250Xi (USA). UV-vis diffuse reflectance spectra (DRS) were measured with a Perkin-Elmer Lambda 950 UV-vis spectrophotometer (USA). Photoluminescence (PL) was performed with an FLS 920 fluorescence spectrophotometer (Edinburgh Instrument, UK). Concentrations of methylene blue were determined by a UV-vis spectrophotometer (TU-1901, Puxi Instruments, China). The photocurrent and electrochemical impedance spectroscopy (EIS) were measured with a CHI 760E electrochemical workstation (Chenhua Instruments, China).

### 2.3 Photocatalytic experiments

For static degradation of methylene blue, we used the device shown in Fig. S1† to evaluate the photocatalytic performance of the CNC framework. First, a CNC framework (0.4 g) with a size of 4 × 4 × 0.5 cm was placed in 100 mL of methylene blue solution with an initial concentration of 10 mg L^−1^. Before the degradation test, the reactor was placed in a dark environment for 1 h to achieve saturated adsorption. Subsequently, the photocatalytic degradation experiment was carried out under visible light irradiation (Xe lamp, 300 W), and 3 mL of the solution was taken from the reactor at regular intervals. The concentration of methylene blue was calculated by monitoring the absorbance of the solution at 664 nm by a UV-visible spectrophotometer. The calculation formula for the photodegradation efficiency of methylene blue is provided in the ESI.† In the reusability experiment, after 3 h of photocatalysis test, the CNC framework was washed repeatedly with deionized water, and then used in the next cycle.

In order to study the degradation ability in the dynamic state, we fixed the CNC frame in the middle of the self-made dead-end filter (Fig. S2†), and then poured the methylene blue solution with the initial concentration of 3 mg L^−1^. During the experiment, the height of the liquid level was always kept at 20 cm, so that a low driving pressure of about 2 kPa was generated, allowing the liquid to pass through the CNC framework under gravity. Then, photocatalytic degradation was carried out under visible light irradiation, and 3 mL of effluent was taken at intervals for degradation efficiency analysis.

## 3 Results and discussion

### 3.1 Materials characterizations

**Fig. 2:**
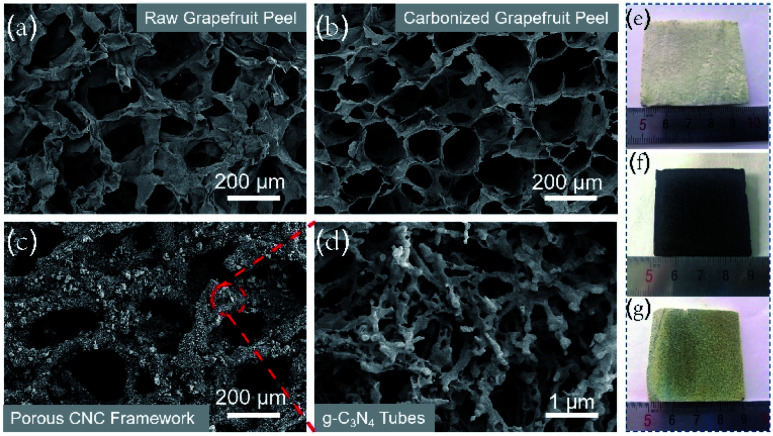
SEM images of raw grapefruit peel (a), carbonized grapefruit peel (b), porous CNC-12 framework (c) and magnified picture (d), and photographs of their corresponding samples (e–g).

The freeze-dried grapefruit peel had a white appearance and porous network structure with a pore size of 120–180 μm (Fig. 2a and e). During the calcination process, although the lignin and cellulose of the grapefruit peel was carbonized at high temperature, the original porous framework is still maintained (Fig. 2f). At the same time, the original wall thickness of 100 ± 10 μm was reduced to 10 ± 1 μm, thus forming a thinner sheet-like inner wall and increasing the pore size (Fig. 2b). The gas and water vapor generated at high temperature penetrate the inner wall in the process of outward diffusion, leaving several holes in the wall.

After CVD treatment, a large amount of g-C_3_N_4_ deposited on the inner wall of the carbonized frame, and the black carbonized framework became dark yellow (Fig. 2c and g). The enlarged SEM image showed that g-C_3_N_4_ had a hollow tubular structure (Fig. 2d), and it can be also proved from the TEM image (Fig. S3†). The possible reason for the unique structure is that in the CVD process, urea is converted to cyanuric acid and melamine at about 400 °C. Then the two products form complex supramolecular melamine–cyanuric acid nanorods through hydrogen bonding. Subsequently, the supramolecular composite nanorods are transformed into tubular g-C_3_N_4_ at 550 °C.^33^ When the urea content is 10 g, the tubular g-C_3_N_4_ on the CNC-10 frame appears incomplete (Fig. S4a†). When the urea content is increased to 12 g, g-C_3_N_4_ presents a radial tube shape with a diameter of 100–200 nm and a length of 700–800 nm. At the same time, pores can be observed on the tube wall (Fig. 2d). As the urea content continued to increase, the tubular g-C_3_N_4_ gradually grew longer. Finally, when the urea content is 14 g, the tubes tend to stick together without obvious pores (Fig. S4b†).

**Fig. 3:**
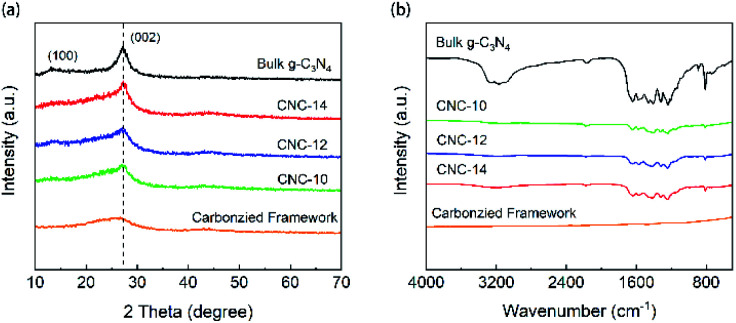
XRD patterns (a) and FTIR spectra (b) of bulk g-C_3_N_4_, carbonized framework and CNC frameworks with different usages of urea.

Fig. 3a shows the XRD patterns of the bulk g-C_3_N_4_ and porous CNC frameworks. For the carbonized grapefruit peel, it has broad diffraction peaks at 26.1° and 43.3° due to the formation of amorphous carbon.^33^ The XRD pattern of the bulk g-C_3_N_4_ has two peaks at 13.1° and 27.3°, corresponding to (100) and (002) crystal planes, respectively. It is generally believed that the (002) crystal plane of g-C_3_N_4_ is related to the interlayer reflection of the graphene-like structure, while the (100) crystal plane is derived from the aromatic repeating unit in the plane.^34^ Compared with g-C_3_N_4_, the diffraction peak intensity of CNC frameworks on the (100) crystal plane becomes less obvious. The three samples of CNC-10, CNC-12 and CNC-14 frameworks all have peaks at 27.3°, and the peak intensity increases with the increase of g-C_3_N_4_ loadings.

The FTIR spectra of Fig. 3b show the surface functional groups of the carbonized framework, CNC frameworks and bulk g-C_3_N_4_. The characteristic absorption peaks of the bulk g-C_3_N_4_ and the CNC frameworks are almost the same. The absorption peak at 2900–3500 cm^−1^ originates from the stretching vibration of N–H and O–H. The absorption band at 1200–1700 cm^−1^ corresponds to the C–N tensile vibration of the aromatic heterocyclic ring. And obvious absorption peaks can be observed at 1312 cm^−1^ and 1236 cm^−1^, which correspond to the tensile vibrations of N–(C)_3_ and C–NH–C of the bulk g-C_3_N_4_. In terms of CNC frameworks, they have a weaker absorption peak intensity at 1500–1700 cm^−1^ than the bulk g-C_3_N_4_.

The reason is that the tubular g-C_3_N_4_ on the CNC framework has a nano-sized structure, and the corresponding nitrogen heterocyclic structure is less, leading in lower absorption peak intensity of the nitrogen heterocyclic ring.^35^ However, as the deposited g-C_3_N_4_ increases, the intensity of the IR absorption peak of the CNC frameworks becomes stronger, indicating that long tubular g-C_3_N_4_ with more nitrogen heterocyclic structure may be formed. At the same time, no other characteristic peaks are observed in FTIR spectra, which proves that the presence of the carbon framework does not change the thermal condensation reaction of urea.

**Fig. 4:**
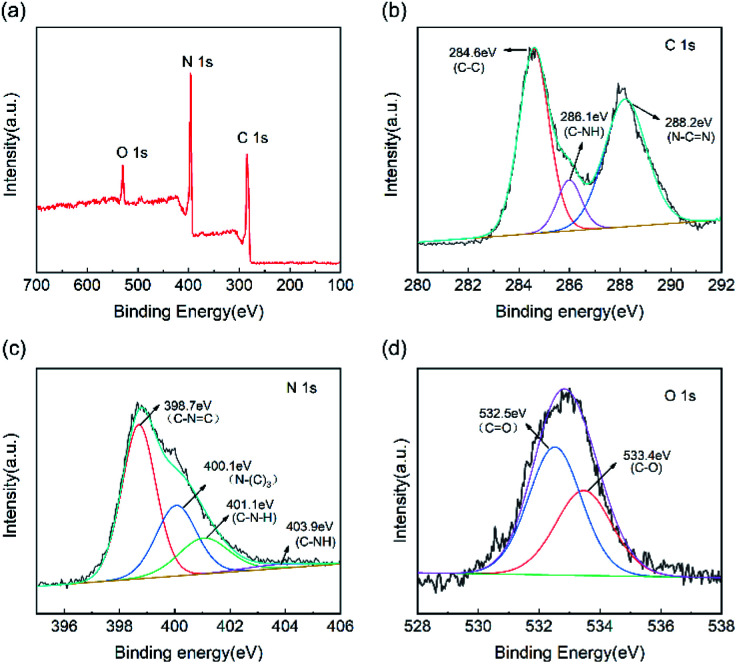
(a) XPS full spectrum of CNC-12 framework and high-precision spectra of (b) C 1s, (c) N 1s and (d) O 1s.

In terms of the chemical composition of the CNC framework, Fig. 4a indicates that C, N, and O element are detected in the XPS spectrum of CNC-12 framework. For the high-precision spectrum of the C 1s (Fig. 4b), it has two typical peaks at 284.6 eV and 288.2 eV, which are attributed to the sp^2^ hybridized C–C bond and N–C=N in the triazine ring.^36^ The peak at 286.1 eV has a large peak area, indicating that the sp^2^ C–C content in CNC-12 is relatively high, which is due to the carbon framework itself. In addition, the peak of C 1s at 288.2 eV corresponds to C–NH, which indicates that CNC-12 contains the C–NH structure of g-C_3_N_4_. The XPS spectrum of N 1s can be divided into four peaks of 398.7 eV, 400.1 eV, 401.1 eV and 403.9 eV (Fig. 4c), which correspond to the sp^2^ hybrid nitrogen (CN=C) in the triazine ring, N–(C)_3_ groups, amino group (C–N–H) originating form the incomplete triazine ring structure under thermal condensation, and π bond excitation, respectively.^37^ O 1s has peaks at 533.4.75 eV and 532.5 eV (Fig. 4d), corresponding to the C=O and C–O bonds, respectively.

### 3.2 Optical and electrochemical properties

**Fig. 5:**
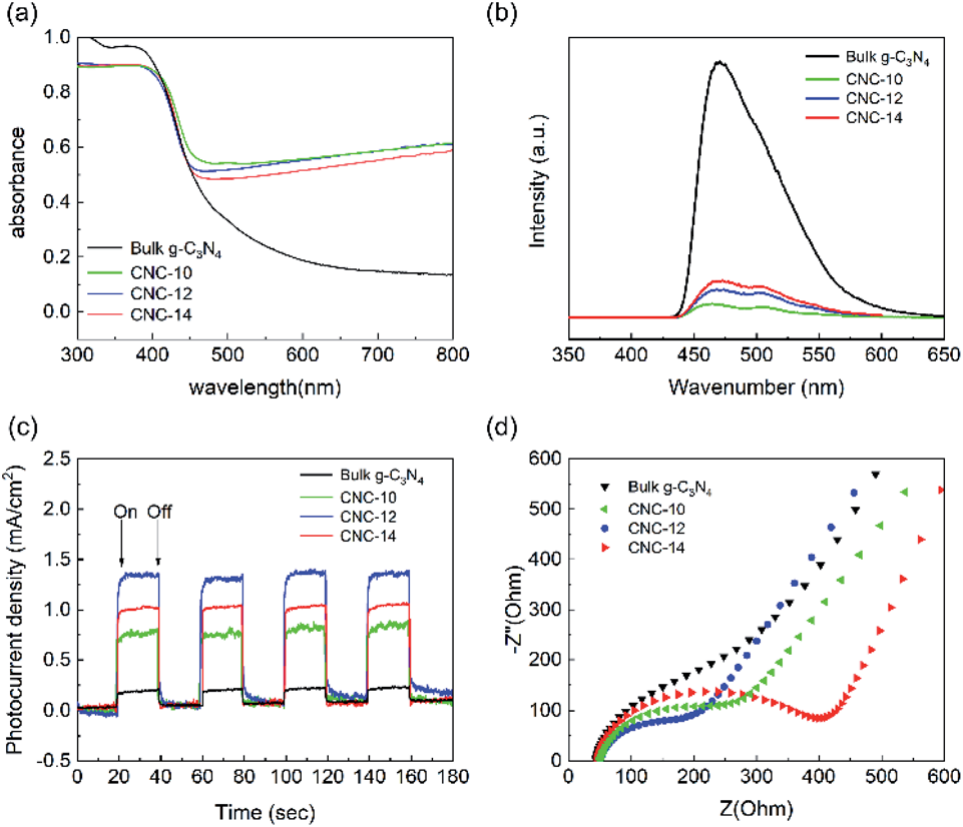
UV-vis absorption spectra (a), photoluminescence spectra (b), instantaneous photocurrent response curves (c) and AC impedance curves (d) of bulk g-C_3_N_4_, CNC-10, CNC-12 and CNC-14.

Fig. 5a shows the UV-vis absorption spectrum of bulk g-C_3_N_4_ and CNC framework. The introduction of the black carbonized framework makes them possess stronger absorption in the entire visible light region. With the increase of the tubular g-C_3_N_4_ content in the framework, the carbonized framework with full-spectrum absorption capacity is gradually covered, resulting in a decrease in the absorbance of the CNC framework in the wavelength range of 500–800 nm. The photoluminescence (PL) spectrum shows that the bulk g-C_3_N_4_ has a strong absorption at 455 nm, which is caused by the direct recombination of electrons and holes generated by light excitation (Fig. 5b). In contrast, all CNC frameworks have weaker fluorescence intensity. The reason is that photogenerated electrons can quickly migrate to the surface of the porous carbonized framework, effectively inhibiting the direct recombination of electrons and holes, thereby reducing the fluorescence intensity.

In addition, we performed photocurrent response tests on bulk g-C_3_N_4_ and three CNC frameworks to evaluate the separation ability of generating carriers and photogenerated electrons. As shown in Fig. 5c, when the lamp is turned on, the photocurrent density rapidly rises to an approximately stable value. When the lamp is off, the photocurrent density quickly drops to zero. The analysis result indicates that the CNC framework has a higher photocurrent density than the bulk g-C_3_N_4_, which means that the recombination of electrons and holes are reduced, and more photogenerated carriers are produced to remove organic pollutants. During the CVD process, as the dosage of urea increases, the amount of tubular g-C_3_N_4_ grown in the CNC-14 framework also increases, resulting in a decrease in the transfer capacity of photogenerated electrons to the carbonized framework. Too low coverage of the tubular g-C_3_N_4_ on the framework will result in a decrease in the number of photogenerated electrons, resulting in low current density of CNC-10 frameworks.

Fig. 5d shows the AC impedance curve, which can be used to study the charge transfer resistance and separation efficiency between photogenerated electrons and holes in the sample. The size of the semicircle of the Nyquist plot is related to the material resistance. The smaller semicircle means that the material has a smaller resistance to electron transmission. The analysis shows that the size of the semicircle of the CNC framework is smaller than that of bulk g-C_3_N_4_, indicating that the porous carbonized framework with a three-dimensional structure can be used as an acceptor to effectively reduce the electron transport resistance, accelerate the interface charge transfer speed, and thus facilitate the separation of electrons and holes generated by light excitation.

### 3.3 Photocatalytic degradation performance

**Fig. 6:**
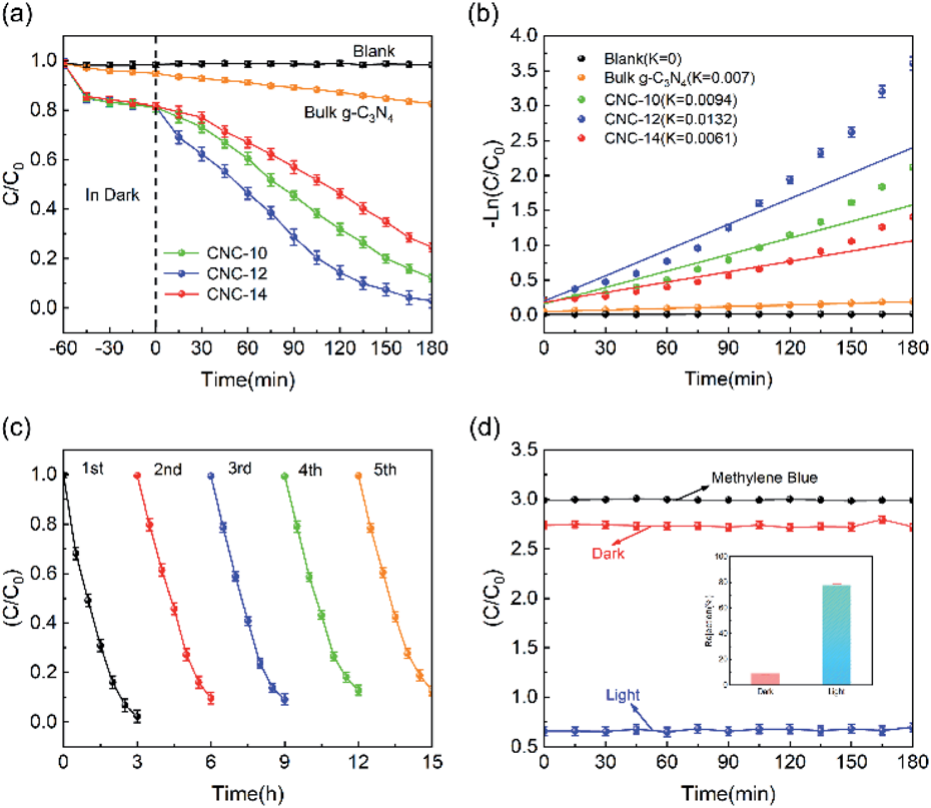
(a) The degradation performance and (b) kinetic linear simulation curves of bulk g-C_3_N_4_ and CNC frameworks; (c) Reusability test of the CNC-12 framework; (d) The removal performance of the CNC-12 framework in the dynamic photodegradation process.

Fig. 6a shows the photocatalytic degradation performance of CNC frameworks in a static state test. Before the photodegradation procedure, the sample was placed into a 100 mg L^−1^ methylene blue solution and performed adsorption in the dark. For the test using CNC frameworks as the photocatalyst, they can adsorb about 20% of methylene blue within 1 h, which is much higher than the control group using bulk g-C_3_N_4_. This is because the porous framework has a higher specific surface area and can absorb more methylene blue. Under visible light irradiation, the blank group and the control group using g-C_3_N_4_ could not effectively remove methylene blue. In contrast, when CNC-12 framework is used as the photocatalyst, nearly 98% of methylene blue can be degraded after 180 minutes, indicating that the sample has excellent photocatalytic activity. When using CNC-10 frameworks, the tubular g-C_3_N_4_ content on the carbonized framework is relatively low, resulting in weaker photodegradability. When CNC-14 framework is used, excessive urea usage will cause the internal structure of tubular g-C_3_N_4_ close to bulk g-C_3_N_4_, so it has the worst photocatalytic ability among the three samples. Fig. 6b shows the analysis of the reaction kinetics of methylene blue degradation by studying the degradation ability of different photocatalysts. The first-order model indicates that the CNC-12 framework has the largest *k* value of 0.0132 min^−1^, which means that its degradation rate is the fastest compared with CNC-10, CNC-14 and bulk g-C_3_N_4_.

The above results are consistent with the transient photocurrent response, which proves that CNC-12 framework has the strongest photocatalytic activity.

In addition, the stability of the photocatalyst is of great significance to its practical application. Therefore, a five-cycle test was performed to examine the reusability of the photocatalyst. As shown in Fig. 6c, after 5 cycles, the photodegradation activity of the CNC-12 framework is not significantly reduced, but the methylene blue could still be removed by about 90%, indicating it has excellent photocatalytic stability. Fig. 6d is the photocatalytic degradation curve of CNC-12 framework driven by gravity. In the dark condition, the CNC-12 framework can absorb about 9% of methyl blue while the solution is flowing. Under visible light, although the contact time between the flowing solution and the CNC framework is not as long as that in the static experiment, the photocatalytic degradation rate can still reach about 77.65% due to fast adsorption and degradation kinetics, which indicates that the CNC framework can have the dual functions of membrane filtration and photocatalysis.

### 3.4 Mechanism of photocatalytic degradation

Typically, disodium ethylenediamine tetraacetate (EDTA-2Na, 10 mM), isopropanol (IPA, 10 mM) and *p*-benzoquinone (BQ, 10 mM) can be used to trap the holes, ˙OH radicals and ˙O^2−^ radicals, respectively. In the radical species capture experiment of CNC-12, different scavengers were added into the MB solution to investigate the role of holes, ˙OH radicals and ˙O^2−^ radicals. As shown in Fig. S5,† by the addition of BQ and IPA, the photocatalytic degradation efficiency of MB was 43% and 46%, respectively, which was significantly lower than the efficiency of EDTA-2Na (77%) and no-trapped agent (98%). The above analysis results indicate that ˙O^2−^ radicals and ˙OH play a major role in the photocatalytic degradation of MB.

**Fig. 7:**
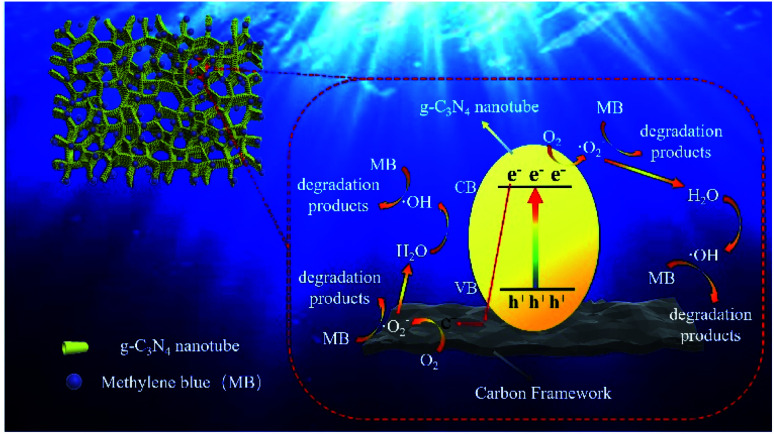
Mechanism diagram of photocatalytic degradation ability of the porous CNC framework.

The possible mechanism of MB degradation was proposed in Fig. 7. Under visible light irradiation, the electrons on the g-C_3_N_4_ nanotubes transition from valence band to conduction band, resulting in holes in valence band. Carbon materials had excellent electron transport capabilities which can effectively transfer photogenerated electrons. Therefore, the carbon frameworks promoted the effective separation and transfer of photogenerated electrons and holes. The electrons in the conduction band of g-C_3_N_4_ could reduce O_2_ to ˙O^2−^, and at the same time, the ˙O^2−^ in valence band could oxidize water molecules to ˙OH. Afterwards, ˙OH and superoxide radicals can synergistically oxidize the MB to CO_2_ and H_2_O (Fig. S6†), *etc.*^38^ Photocatalytic degradation is mainly divided into two steps: (1) first, the hydrophilic porous network framework adsorbs pollutant molecules in the water to the inner wall and pores; (2) under visible light irradiation, the tubular g-C_3_N_4_ will be excited to generate electrons, which are then transferred to the carbonized framework, thereby generating active free radicals, and finally achieving the degradation of pollutants. In this study, the efficient photocatalytic degradation efficiency is attributed to the following reasons: (1) CNC frameworks prepared by CVD method can effectively reduce the size of g-C_3_N_4_ to form nano-sized tubes, thereby increasing the photocatalytic performance; (2) the excellent adsorption capacity of the carbonized framework can continuously enrich pollutants on the surface of the CNC framework; (3) because the carbonized framework has excellent electron transport capabilities, they can effectively separate photogenerated electrons and improve light absorption range and catalyst activity. It can be seen that under the joint promotion of adsorption and photocatalytic degradation, the CNC framework can efficiently remove methylene blue from water, thereby achieving water purification.

## 4 Conclusions

In this paper, a low-cost porous composite framework with visible light catalytic ability was prepared by the CVD method. The carbonized grapefruit peel is used as a support carrier, and nano-sized tubular g-C_3_N_4_ is polymerized on it, thereby increasing the active sites of the CNC framework. The carbonized framework can enhance the light absorption of the catalyst and effectively promote the separation of electron–hole pairs, thereby improving the photocatalytic ability under visible light irradiation. In addition, the CNC framework also shows excellent photocatalytic activity in both static and dynamic photocatalysis. The photocatalytic cycle experiment proves that the tubular g-C_3_N_4_ can be stably loaded on the porous carbonized framework and has good stability in the long-term operation. Therefore, the CNC framework has great potential in the photocatalytic degradation of water-soluble dyes.

## Conflicts of interest

The authors declare that they have no known competing financial interests or personal relationships that could have appeared to influence the work reported in this paper.

## Supplementary Material

RA-011-D1RA02918E-s001
