# Supplementary material for: Tubular g-C3N4/carbon framework for high-efficiency photocatalytic degradation of methylene blue
Source: RSC Adv. 2021 May 21;11(30):18519–24. doi: 10.1039/d1ra02918e (PMC9033409; doi:10.1039/d1ra02918e)
Supplement: RA-011-D1RA02918E-s001 [file RA-011-D1RA02918E-s001.pdf]

Electronic Supplementary Material (ESI) for RSC Advances.
This journal is © The Royal Society of Chemistry 2021

Supporting Information

Tubular g-C_3N_4/Carbon Framework for High-efficiency Photocatalytic Degradation of Methylene Blue

Hai-cheng Li[1], Lin-lin Zang[2], Feng-tong Shen[1], Li-bin Wang[1], Li-guo Sun[*,1], Fu-long Yuan[*,1]

[1] School of Chemical Engineering and Materials, Heilongjiang University, Harbin 150080, P. R. China.

[2] State Key Laboratory of Urban Water Resource and Environment, School of Environment, Harbin Institute of Technology, Harbin 150090, P. R. China.

[*]Corresponding Authors:

E-mail: sunliguo1975@163.com (L. Sun); yuanfulong@hlju.edu.cn

Experiment

Degradation efficiency formula:

The degradation efficiency of methylene blue is calculated by the following formula:

$$Efficiency = \frac{C_0 - C}{C_0} * 100\%$$

C_0 is the concentration of the initial methylene blue solution, and C is the concentration of the methylene blue solution tested with a spectrophotometer.

Results and discussion

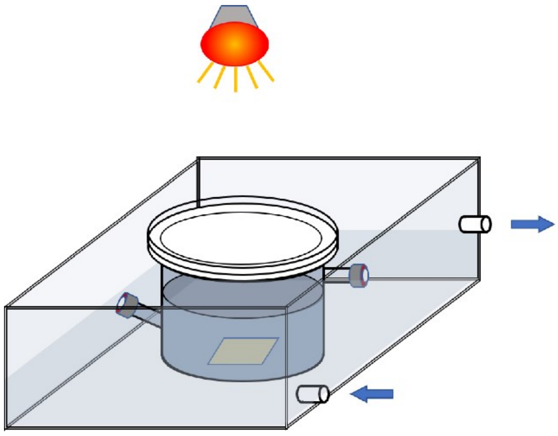

Fig. S1. Schematic diagram of the static photocatalysis process in a water tank.

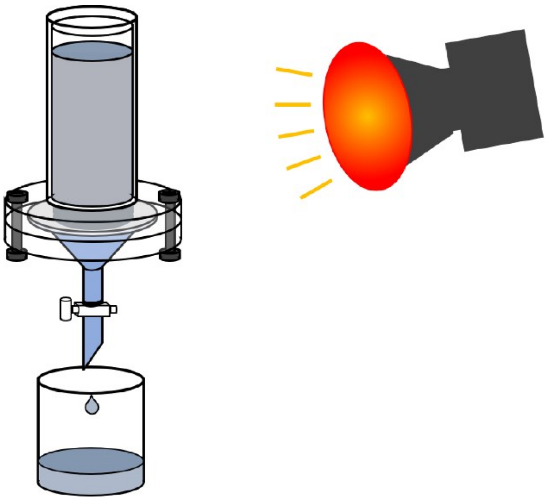

Fig. S2 Schematic diagram of the dynamic photocatalysis process.

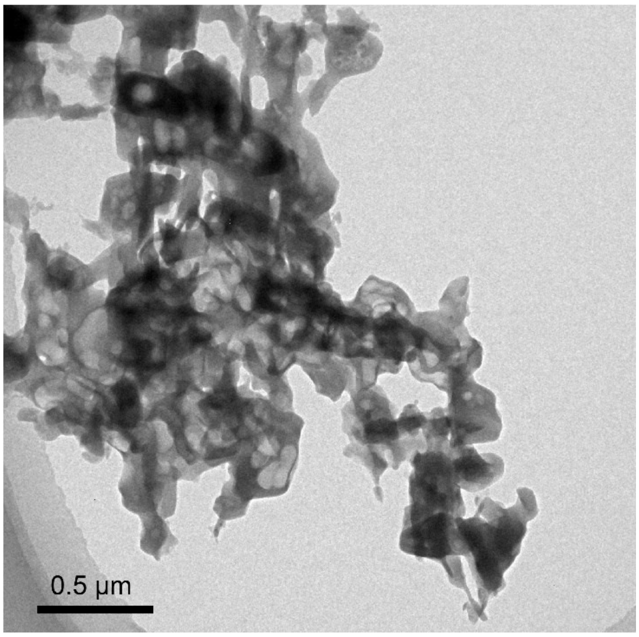

Fig. S3 TEM images of CNC-12.

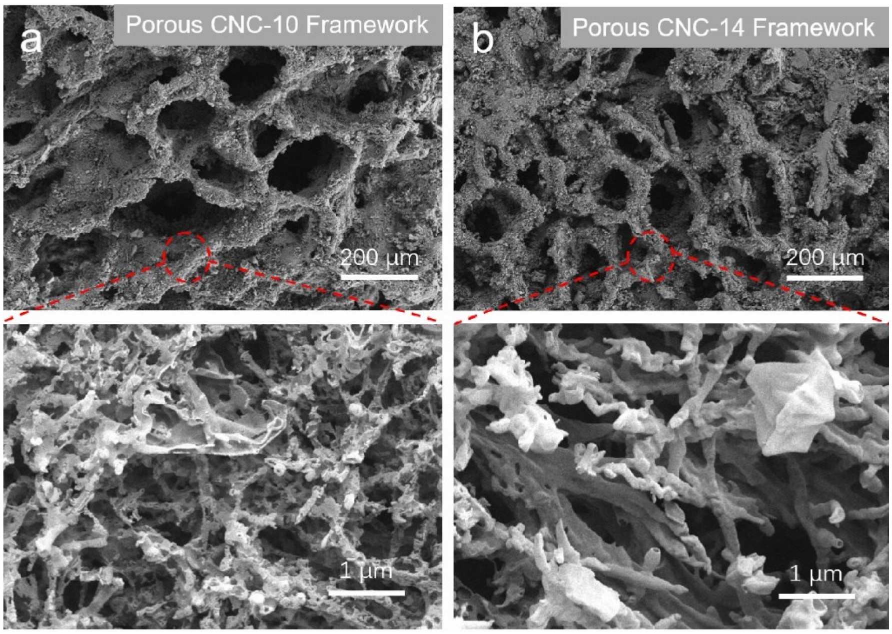

Fig. S4 SEM images of CNC-10 (a), and CNC-14 (b).

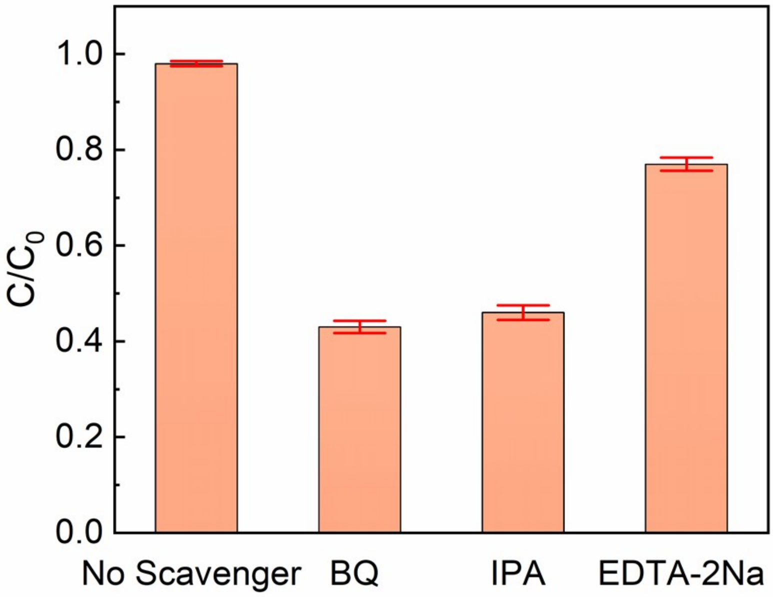

Fig. S5 Radical species capture experiment.

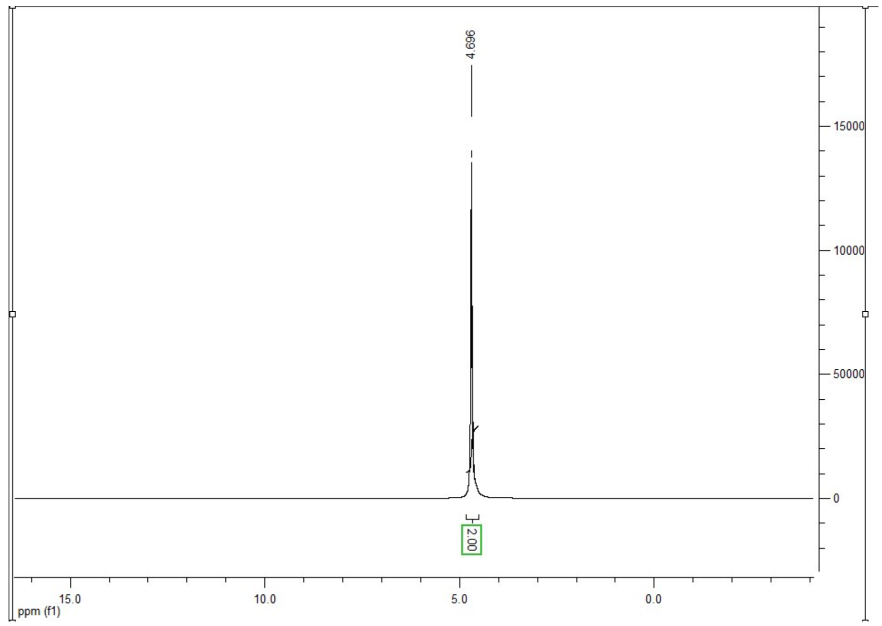

Fig. S6 1H-NMR (400 MHz, D_2O) δ 4.696 (m, J = 4.696, 2H)
